# The role of metformin in tuberculosis control among TB and diabetes mellitus comorbid individuals

**DOI:** 10.3389/fmicb.2025.1549572

**Published:** 2025-04-25

**Authors:** Phillip Tetteh, Emelia K. Danso, Stephen Osei-Wusu, Dorothy Yeboah-Manu, Prince Asare

**Affiliations:** Noguchi Memorial Institute for Medical Research, University of Ghana, Accra, Ghana

**Keywords:** *Mycobacterium tuberculosis*, metformin, host-directed therapeutics, diabetes, TB-diabetes patients, immunomodulatory

## Abstract

Tuberculosis (TB) is a major global health concern, and its control is particularly hindered in patients with comorbidities such as type 2 diabetes mellitus (TBDM). Metformin, a commonly prescribed antidiabetic medication, has gained attention because of its potential role in improving TB treatment outcomes in patients with TBDM. This review aims to assess the role of metformin in TB case management among TBDM comorbid individuals. By reviewing the available literature, we aimed to explore the potential benefits, complications, mechanisms, and considerations surrounding metformin use as an adjunctive therapy for TB treatment. The findings of this review will shed light on current understanding and highlight areas for further investigation.

## 1 Introduction

Tuberculosis (TB) is an infectious disease that most often affects the lungs and is caused by the *Mycobacterium tuberculosis* complex (MTBC). It is an ongoing global health threat and in 2023, the World Health Organization (WHO) estimated that 10.8 million people worldwide developed TB, and approximately 1.25 million died of the disease (World Health Organization, 2024). Although not everyone who is infected with any of the members of the MTBC develops the disease, those with diabetes and other risk factors, such as HIV, smoking, alcohol use disorders, and malnutrition, are at a greater risk of active infection (World Health Organization., 2023b).

The main current control measures include early diagnosis and treatment. The standard treatment regimen for drug-susceptible TB consists of a six-month course of first-line anti-TB drugs, including a two-month intensive phase with four antibiotics (isoniazid, rifampicin, pyrazinamide, and ethambutol), followed by a four-month continuation phase with isoniazid and rifampicin (World Health Organization, 2022b). The updated WHO guidelines recommend all-oral, shorter, and more effective treatment regimens for most patients with drug-resistant TB (DR-TB). These regimens typically last 6–9 months and include bedaquiline, pretomanid, linezolid, and moxifloxacin (World Health Organization, 2022b).

In addition to pharmacological treatment, TB control measures include active case finding, contact tracing, and ensuring that patients complete their treatment through directly observed therapy (DOT) (World Health Organization, 2017). Additionally, for latent TB infection, the WHO also emphasizes the importance of preventive therapies with isoniazid, rifampin, or combinations of isoniazid plus rifampin or rifapentine to prevent the progression to active disease (World Health Organization, 2020). Also, the Bacillus Calmette-Guérin (BCG) vaccine is the only licensed vaccine for tuberculosis (TB) and has been widely used for TB control, particularly in high-burden countries. BCG is effective in preventing severe forms of TB, such as miliary TB and TB meningitis in children, but its protection against pulmonary TB in adults is variable (Li et al., 2022). One of the main limitations of BCG is its waning immunity over time, necessitating the need for booster strategies or novel vaccines (Andersen and Scriba, 2019). Investigating host-directed therapies, such as metformin, in conjunction with BCG could improve TB control by modulating host immune responses (Singhal et al., 2014).

Diabetes mellitus (DM) is a chronic metabolic disease characterized by elevated blood glucose levels, that may lead to serious damage to the heart, blood vessels, eyes, kidneys, and nerves. The most common type is type 2 diabetes, usually in adults, which occurs when the body becomes resistant to insulin or does not produce enough insulin (World Health Organization, 2023a). According to the International Diabetes Federation (IDF), the number of people living with diabetes has more than tripled since 2000, from 151 million to 537 million (International Diabetes Federation, 2023; Lee et al., 2019).

Management of type 2 diabetes involves lifestyle modifications, such as diet and exercise (Uusitupa et al., 2019), alongside pharmacological interventions. These include metformin as a first-line therapy that improves insulin sensitivity and reduces glucose production in the liver. Other options include sulfonylureas, which increase insulin secretion, SGLT2 inhibitors, which reduce glucose reabsorption in the kidneys, and GLP-1 receptor agonists, which enhance insulin secretion and promote satiety. Insulin therapy may also be necessary for some patients, particularly based on individual patient needs and responses (Aschner, 2017). Additionally, the IDF recommends patient education and support, regular follow-ups, and personalized care to achieve and maintain glycemic control, prevent complications, and improve overall quality of life (Aschner, 2017).

DM leads to starvation of immune cells, thus affecting immune function, and predisposing the affected to infection with several infectious diseases such as TB through mechanisms such as priming of neutrophils leading to excessive neutrophil extracellular trap (NET) formation. While NETs trap pathogens, their overproduction can exacerbate lung inflammation and tissue damage (Shrestha et al., 2024) thus several observational studies have supported the notion that DM is a risk factor for developing TB, necessitating good glycemic management (Park et al., 2019). DM is linked to a two to threefold increased risk of developing TB, a twofold higher risk of death during TB treatment, a fourfold higher risk of TB relapse after treatment completion, and a twofold increased risk of DR-TB. In 2020, an estimated 370,000 new TB cases were attributed to diabetes (World Health Organization, 2021). In 2019, more than 15% of TB patients globally were estimated to have diabetes. This translates to about 1.5 million people with both TB and diabetes, necessitating coordinated care and follow-up to optimize the management of both conditions (World Health Organization, 2021). The simultaneous initiation of TB treatment and DM management in TBDM patients is aimed to improve TB treatment outcomes and reduce DM-related morbidity and mortality. Given the increasing prevalence of TB-DM co-infection and the growing body of evidence supporting host-directed therapies (HDTs), exploring metformin’s potential is critical. Studies indicate that TB-DM patients experience higher mortality rates, slower treatment responses, and increased drug resistance risk, highlighting the need for adjunctive therapies to improve outcomes (Singhal et al., 2014; World Health Organization, 2021). Moreover, metformin’s well-documented immunomodulatory properties and its accessibility as an affordable, widely available drug make it a promising candidate for TB treatment (Fatima et al., 2021; Zumla et al., 2013). By reviewing the available literature (summarized in Table 1), we aimed to explore the current understanding of metformin’s role in managing TB-DM comorbidity and discuss its implications for improving patient outcomes.

**TABLE 1:** Summary of key studies evaluating metformin’s effects on TB treatment outcome.

References	Study type	Strength	Weakness	Specific results	Quality of evidence
Singhal et al., 2014	Preclinical (mouse model)	Mechanistic insights into ROS/autophagy and a controlled experimental design.	Animal model; no human data.	Metformin enhances ROS production and autophagy, improving bacterial clearance (Singhal et al., 2014).	Low (Preclinical, animal study; no human translation).
Lee et al., 2018	Retrospective cohort	Real-world data in cavitary TB-DM patients with clinically relevant outcomes.	Retrospective design; unadjusted confounders (e.g., glycemic control).	Metformin enhanced sputum culture conversion in cavitary pulmonary TB patients with high bacterial loads (OR, 10.8; 95% CI, 1.22–95.63) (Lee et al., 2018).	Low-moderate (Observational; retrospective design).
Ma et al., 2018	Longitudinal study	Long-term follow-up (3 years) with a focus on relapse rates.	Observational; potential selection bias.	Over a 3-year follow-up, relapse rates were 6.3% (1/16) in the metformin group and 35.7% (15/42) in the non-metformin group (*P* = 0.045) (Ma et al., 2018).	Moderate (Observational; longitudinal but lacks randomization).
Lachmandas et al., 2019	Preclinical (Human PBMCs)	Human cell-based mechanistic insights with measured cytokine profiles.	*In vitro* model with a small sample size.	Metformin enhanced glycolysis and inhibited mTOR signaling in PBMCs, reducing pro-inflammatory cytokines while boosting phagocytosis (Lachmandas et al., 2019).	Low (Preclinical; *in vitro* human cells).
Mishra et al., 2022	Prospective study	Prospective design; controlled for glycemic levels	Single-center.	Metformin use significantly increased sputum smear conversion (*p* = 0.0318, unpaired *t*-test) compared to non-users (Mishra et al., 2022).	Moderate (Prospective cohort; lacks randomization).
Wang et al., 2022	Observational Study	Large sample (*n* = 927) and a focus on smear conversion.	Confounding (e.g., DM management). Observational design.	56% smear-negative conversion (metformin) vs. 27% (non-users) (Wang et al., 2022).	Low (Observational; retrospective with unmeasured variables).
Padmapriydarsini et al., 2022	Randomized Clinical Trial (RCT)	RCT design; controlled for confounders.	A small sample with a short follow-up.	After 8 weeks of anti-TB therapy, metformin users showed significantly fewer cavitary lesions on X-ray (5.3% vs. 12.9%; RR, 0.42; 95% CI, 0.18–0.96; *P* = 0.041) and lower inflammatory markers (Padmapriydarsini et al., 2022).	High (RCT; limited by sample size but randomized and controlled).

## 2 Immunomodulatory agents as host-directed therapy in tuberculosis treatment

Preclinical and clinical research has uncovered numerous immunomodulatory agents that have improved TB therapies in recent years (Zumla et al., 2016). The application of immunomodulatory agents as HDT for TB treatment has gained significant attention for its potential to modulate the host’s immune responses against TB, increasing the clearance of *Mycobacterium tuberculosis* (Mtb) in immune cells, minimizing tissue damage, and reducing relapse (Table 2). Several of these agents have already been approved for the treatment of other diseases, whereas others are undergoing clinical trials (Zumla et al., 2016). These include metformin, loperamide, TNF-α Inhibitors, etc (Juárez et al., 2018; Singhal et al., 2014; Young et al., 2020). The GRADE (Grading of Recommendations, Assessment, Development, and Evaluations) framework (Prasad, 2024) was applied to evaluate the reliability of evidence supporting these immunomodulatory therapies. This tool systematically assesses the quality of evidence and the strength of recommendations by considering factors such as study design, risk of bias, consistency, and clinical relevance. Utilizing GRADE in the context of immunomodulatory agents ensures that TB treatment strategies are informed by robust and evidence-based decision-making.

**TABLE 2:** Immunomodulatory agents as adjunct therapy for tuberculosis treatment.

Name of drug	Intended usage	Adjunct therapeutic effect against TB	Quality of evidence
Metformin	Treatment of type-2 diabetes	Restricts the growth of mycobacteria through the induction of mitochondrial production of reactive oxygen species (Singhal et al., 2014).	Low: due to confounding in observational data, variable dosing, and inconsistent populations.
Loperamide	Diarrheal treatment	It increases the ability of macrophages to clear Mtb by stimulating the reduction of TNFα, which also reduces tissue damage (Juárez et al., 2018).	Very low: due to lack of human trials; limited to macrophage assays with no clinical relevance to TB patients.
Statins	Treatment of cardiovascular and hyperlipidemic diseases	Statins are known to prevent the uptake of Mtb into host cells which is essential for Mtb survival by inhibiting phagocytosis (Fatima et al., 2021).	Low: confounding (e.g., cardiovascular comorbidities); focus on phagocytosis inhibition without randomized control trials (RCTs).
NSAIDs	Anti-inflammatory agents	Mitigates the inflammation resulting from the influx of monocytes, lymphocytes, and neutrophils (Fatima et al., 2021).	Low: due to inconsistent inflammation outcomes; scanty TB-specific human data.
Salazosulfapyridine	Management of rheumatoid arthritis	It stimulates the Mtb clearance of macrophages by inducing the activation of NADPH oxidase which leads to the oxidation of mycothiol within Mtb-infected macrophages, rather than through the production of the metabolite 5-aminosalicylic acid (Mi et al., 2024).	Very low: due to lack of clinical trials; purely *in vitro* mechanistic exploration.
Verapamil	Treatment of cardiovascular diseases and high blood pressure	It disrupts the membrane function of Mtb and induces a membrane stress response. Also, it increases the efficacy of standard anti-TB drugs (Chen et al., 2018).	Low: due to lack of human trials; limited to membrane disruption assays.
Chicoric acid	Treatment of liver disorders	Enhances the cell surface expression of HLA-DR and CD14 molecules on macrophages and increases the production of nitric oxide in macrophages thus preventing the growth of Mtb within macrophages (Abd-Nikfarjam et al., 2018).	Very Low: no clinical or animal validation.
Corticosteroids	Treatment of inflammatory disorders	Reduces inflammation in TB patients with complications like TB meningitis and pericarditis by modulating inflammatory mediator function, suppressing the humoral immune response, and inhibiting leucocyte infiltration to the site of infection (Young et al., 2020).	Moderate: benefits confined to severe inflammation (e.g., pericarditis); small trial sizes.
TNF-α Inhibitors	Anti-inflammatory agents and immunosuppressants.	Increase Mtb susceptibility and exposure to the standard anti-TB drugs by disrupting and penetrating granulomas to expose Mtb bacilli to the anti-TB drugs (Young et al., 2020).	Moderate: increased TB reactivation risk in immunocompromised patients; not tested as direct TB adjuncts.
Vitamin D	Management of autoimmune diseases like multiple sclerosis and rheumatoid arthritis	Stimulates the production of antimicrobial peptides (cathelicidin and defensins), that directly kill Mtb by disrupting the bacterial membranes and enhancing phagolysosome formation in macrophages (Papagni et al., 2022).	Low to moderate: variable antimicrobial peptide effects; small, diverse cohorts.

For individuals with newly diagnosed type-2 diabetes (T2DM), metformin and sulfonylureas are the most often given anti-DM medications worldwide but due to metformin’s tolerably controllable side-effect profile, it serves as the first-line medications for the majority of patients with T2DM and also, the first-choice oral glucose-lowering drug for TB patients (Patil et al., 2019).

### 2.1 Antidiabetic and antimicrobial activity effect of metformin

Metformin lowers blood glucose by activating the 5′-adenosine monophosphate-activated protein kinase (AMPK) which prevents the production of glucose (gluconeogenesis) in the liver and improves the uptake of glucose by muscle cells (Tseng, 2018).

**FIGURE 1:**
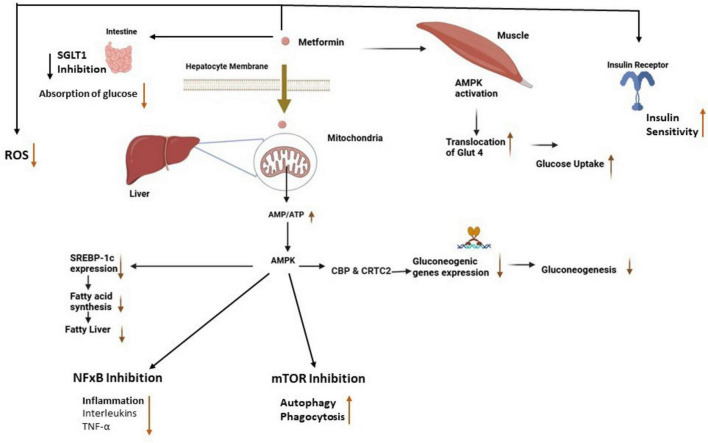
Antidiabetic and antimicrobial effects of metformin. The absorption of metformin into hepatocytes and muscle cells leads to the activation of AMPK, which reduces fatty acid synthesis and gluconeogenesis in the liver while enhancing glucose uptake in muscle cells (Szymczak-Pajor et al., 2022). Activated AMPK inhibits NF-κB and mTOR which leads to reduced inflammation and an increase in autophagy respectively. Also, metformin reduces intestinal glucose absorption, increases insulin sensitivity, and reduces ROS production. AMPK, adenosine monophosphate-activated protein kinase; SREPB-1, sterol-regulatory-element-binding-protein-1; CBP & CRTC2, creb binding protein and creb-regulated transcription coactivator 2, mTOR, mammalian target of rapamycin, NF-κB, nuclear factor kappa-B; ROS, reactive oxygen species; SGLT 1, sodium-glucose cotransporter 1; GLUT 4, glucose transporter type 4.

Reduction of hepatic glucose production: Absorption of metformin into hepatocytes increases the AMP/ATP ratio, leading to the activation of AMPK. Activated AMPK inhibits the expression of gluconeogenic genes such as phosphoenolpyruvate carboxykinase (PEPCK) and glucose-6-phosphatase (G6Pase) by interfering with transcription factors and co-activators, including CREB-regulated transcription coactivator 2 (CRTC2) which reduces the liver’s ability to produce glucose, and directly lowers blood glucose levels (Figure 1) a TB risk factor, as confirmed in mechanistic studies by Foretz et al. (2014). Additionally, activated AMPK by metformin suppresses the expression of sterol regulatory element binding protein-1c (SREBP-1), an important lipogenic transcription factor, which leads to the suppression of fatty acid synthesis in the liver (Kaneto et al., 2021).

Improvement of insulin sensitivity: Metformin improves insulin sensitivity in peripheral tissues like muscle by activating AMPK which stimulates the translocation of glucose transporter type 4 (GLUT4) to the cell surface. This process enhances glucose uptake into the cells (Kaneto et al., 2021), supporting immune cell energy needs. Additionally, metformin improves insulin signaling pathways by reducing the accumulation of intracellular lipids that interfere with insulin receptor signaling as well as reducing macrophage lipid metabolism, potentially starving TB of lipids. It suppresses lipogenesis (fat synthesis) in the liver and muscle tissues, which reduces the levels of free fatty acids and lipid intermediates, such as diacylglycerol (DAG) and ceramides, known to induce insulin resistance. By reducing these lipid intermediates, metformin helps restore the insulin receptor’s ability to respond to insulin, thereby improving glucose uptake (Russell et al., 2019; Zabielski et al., 2018).

Inhibition of intestinal glucose absorption: Metformin also exerts its glucose-lowering effects in the intestines by inhibiting sodium-glucose cotransporter 1 (SGLT1) which is responsible for the uptake of glucose from the intestinal lumen into enterocytes. The inhibition of SGLT1 reduces the amount of glucose absorbed into the bloodstream after a meal, thereby lowering postprandial glucose levels (Cheng et al., 2024).

Although not fully understood, research indicates that metformin can overcome bacterial resistance by modulating the host’s immune response to the infection, enhancing the effectiveness of antibiotics, and increasing the intracellular accumulation of antibiotics by disrupting bacterial outer membranes (Masadeh et al., 2021). According to Masadeh et al. (2021), metformin enhances the effectiveness of antibiotics by disrupting the outer membrane of bacteria, facilitating greater penetration of antibiotics and accumulation within bacterial cells. This mechanism increases the intracellular concentration of antibiotics, thereby improving their efficacy (Masadeh et al., 2021). In a study by Valadbeigi et al. (2023), they found that the combination of metformin and amoxicillin had a strong synergistic effect against *H. pylori* and decreased early *H. pylori* complications, particularly gastritis, bacterial colonization, and inflammation. Additionally, the combination of metformin with amoxicillin reduced the effective dose of the antibiotics needed for the complete eradication of *H. pylori* (Valadbeigi et al., 2023). Metformin has been shown to decrease biofilm formation, increase bacteria sensitivity to oxidative stress, and inhibit the virulence of bacteria (Abbas et al., 2017).

Metformin enhances the immune response to TB by modulating innate and adaptive immunity, offering potential as an adjunctive therapy. In macrophages, it increases phagocytosis and autophagy, a cellular process that degrades and recycles damaged organelles and pathogens via AMPK activation, improving TB clearance. This effect is mediated through the inhibition of mammalian targets of rapamycin (mTOR) By promoting autophagy, metformin helps in the clearance of Mtb from infected macrophages, thereby limiting the spread of the bacteria within the host. This mechanism is particularly important in the context of TB, as the pathogen has evolved strategies to evade immune detection and persist within host cells (Lachmandas et al., 2019; Singhal et al., 2014; Zumla et al., 2013). This was demonstrated in human studies where metformin upregulated genes involved in ROS production and phagocytosis after TB stimulation (Lachmandas et al., 2019). Additionally, mycobacteria enter the host cells through phagocytosis and prevent phagosome maturation, enabling their replication within the cell. The maturation of phagosomes is vital for the elimination of pathogens. Metformin stimulates autophagy and enhances phagolysosome fusion within host cells leading to pathogen elimination (Abniaya et al., 2020).

For adaptive immunity, metformin induces metabolic programming in the CD8+ T cell compartment, promoting the formation of memory-like CXCR3+ T cells that possess enhanced homing abilities and increased protective potential against Mtb (Böhme et al., 2020; Fatima et al., 2021). It also reduces excessive Th1/Th17 inflammation (e.g., IFN-γ, IL-17) while maintaining protective responses, balancing immunity and pathology in TB models (Lachmandas et al., 2019). In an observational study by Singhal et al. (2014), metformin restricts the growth of mycobacteria by inducing mitochondrial production of reactive oxygen species and reduces the inflammatory response in the lungs by suppressing the secretion of inflammatory-associated genes such as IL-1β, TNF-α, IL-6, MCP-1, CXCL5, and CXCL10 in mice which reduces tissue pathology and accelerates bacillary clearance by increasing the number and percentage of mycobacteria-specific interferon-γ (IFN-γ)–secreting CD8+ T cells. Furthermore, AMPK activated by metformin negatively regulates the nuclear factor-kappa B (NF-κB) pathway which drives pro-inflammatory cytokine production by reducing the phosphorylation and degradation of its inhibitor, IκBα. This prevents the translocation of NF-κB to the nucleus, thereby suppressing the expression of inflammatory genes thus reducing secretion of TNF-α, IL-6, and IL-1β, modulating excessive inflammation, and preventing immune overactivation during infection. This suppression has been observed in human monocytes, where metformin reduces inflammatory responses to microbial stimuli (Lachmandas et al., 2019; Moiseeva et al., 2013; Saisho, 2015). Additionally, metformin inhibits the NLRP3 inflammasome, decreasing IL-1β release by stabilizing mitochondrial function and reducing reactive oxygen species (ROS), a mechanism confirmed in metabolic disease models but applicable to infection-related inflammation (Xian et al., 2021).

## 3 Challenges and limitations in current understanding of metformin’s impact/role in TB treatment

Variability in study designs, treatment regimens, and demographic demographic factors significantly impacts the precision, applicability, and generalizability of research findings on metformin’s efficacy in TB treatment. Study design, such as prospective versus retrospective designs, inconsistent drug regimens, and single versus combination therapies introduce challenges in interpreting results. Studies using observational data may be biased by uncontrolled confounding variables, such as variations in disease severity, healthcare access, and socioeconomic position (Singhal et al., 2014). Similarly, heterogeneity in treatment regimens adds complexity, with factors such as the duration of diabetes at the onset of metformin therapy and the use of other antidiabetic medications potentially skewing outcomes (Tseng, 2018).

Clinical trials exploring metformin’s role in TB treatment have yielded mixed results, largely due to differences in dosage, administration alongside full versus single anti-TB regimens, and patient populations. For instance, a retrospective cohort study by Heo et al. (2021) found that only the highest cumulative doses of metformin provided protection against TB development in patients newly diagnosed with type 2 diabetes patients, while lower cumulative doses did not significantly reduce the incidence of active TB infection. Additionally, different trials have produced different results when metformin was administered in conjunction with a whole regimen versus only one anti-tubercular medication (Padmapriydarsini et al., 2022). Furthermore, since most studies have concentrated on diabetic individuals, generalizability is limited. The effect of metformin may differ dramatically between diabetic and non-diabetic patients with TB. Although emerging research suggests that metformin’s ability to modulate immune responses by enhancing autophagy, reducing inflammation, and promoting T-cell activation benefits even individuals without diabetes (Fatima et al., 2021; Singhal et al., 2014; Zumla et al., 2016), further randomized controlled trials are needed to validate these findings and determine optimal dosing strategies for non-diabetic patients. Complicating matters further, certain TBDM patients also have co-infection with HIV, depression, and other chronic illnesses (such as cardiovascular diseases, kidney diseases, etc.), which can affect how well their TB progresses and how well they respond to therapy (Cáceres et al., 2022) thus metformin’s use may be contraindicated.

Geographic and demographic variations also shape research outcomes. Differences in TB prevalence, healthcare access, socioeconomic conditions, and genetic backgrounds across regions and populations influence both TB incidence and metformin’s effectiveness. Studies in high-TB-burden areas may differ markedly from those in low-incidence regions due to distinct transmission dynamics. Additionally, genetic and ethnic diversity can affect TB susceptibility and therapeutic response, further challenging the generalizability of findings. Together, these factors underscore the need for more standardized, inclusive research to clarify metformin’s role in TB management across diverse contexts (Boadu et al., 2024).

### 3.1 Impact of metformin on TB treatment and its implications for TB control and diabetes management

The Global TB Report highlights the significant challenges of managing the comorbidity in TB and DM. This combination poses serious health risks, as diabetes increases the risk of developing TB two to three times, and individuals with both conditions are more likely to experience poor TB treatment outcomes, including higher mortality rates, increased risk of TB relapse, and greater susceptibility To DR-TB (World Health Organization, 2021). The report underscores the need for integrated, patient-centered care for patients with both TB and diabetes. This involves collaborative actions between TB and diabetes programs, including regular screening for TB in diabetic patients and vice versa, as well as coordinated treatment and management strategies (World Health Organization, 2021). Patients with TB and diabetes may require additional interventions beyond adjustments to their medication regimens. It is recommended that counseling and education be provided to individuals with TB who have recently been diagnosed with diabetes (Riza et al., 2019).

Metformin, a widely used first-line medication for type 2 diabetes, has been investigated for its potential adjunctive role in tuberculosis (TB) treatment, particularly due to its immunomodulatory and anti-inflammatory properties. Research suggests that metformin may enhance various aspects of TB management, particularly in patients with cavitary pulmonary TB, who typically face higher bacterial loads and slower sputum conversion rates. For instance, Lee et al. (2018), reported improved sputum culture conversion rates in a retrospective cohort study of 93 patients with cavitary TB, suggesting metformin’s potential benefit which conflicts with a clinical trial conducted by Padmapriydarsini et al. (2022), stating that the addition of metformin to the anti-TB drugs did not hasten sputum culture conversion. This discordant observation may be due to the differences in metformin dosage administered and the study’s small sample size, lack of adjustment for confounders like disease severity, therapy adherence, or glycemic control, and possible selection bias where metformin patients might have had better healthcare access or milder diabetes limit its conclusions. Similarly, in an observational study conducted by Wang et al. (2022), a significantly greater proportion of TB patients in the smear-negative conversion group received metformin treatment compared to those in the persistently positive group.

**FIGURE 2:**
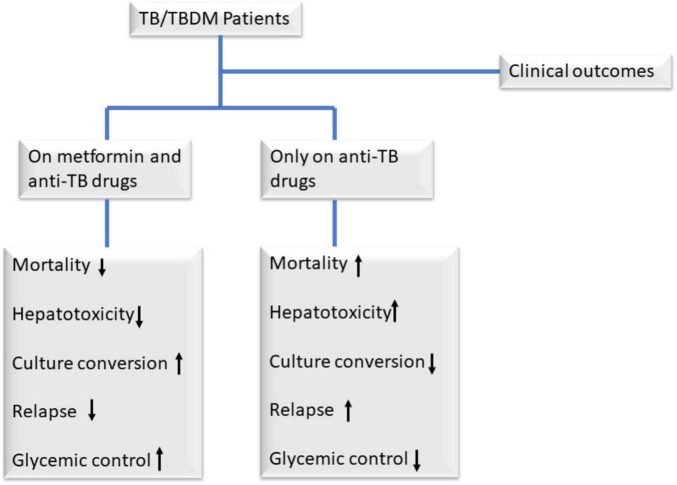
Clinical outcomes among TB/TBDM patients on metformin and anti-TB drugs, and those only on anti-TB drugs. Observational retrospective studies have demonstrated that adding metformin to standard anti-TB therapy offers significant benefits for patients with TB and diabetes mellitus (DM). Compared to those receiving only anti-TB drugs, patients on metformin exhibit reduced mortality, treatment failure, and relapse rates, alongside faster sputum culture and smear-microscopy conversion due to metformin’s ability to inhibit intracellular *Mycobacterium tuberculosis* (Mtb) growth (Degner et al., 2018; Naicker et al., 2020; Singhal et al., 2014). Additionally, metformin accelerates lung cavity resolution on chest X-rays and improves glycemic control, addressing key challenges in TB-DM management (Singhal et al., 2014).

Additionally, the use of metformin as a combination therapy with existing anti-TB drugs has been shown in preclinical studies to facilitate early sputum culture conversion (Figure 2) by inhibiting the intracellular growth of Mtb through enhanced phagocytosis and reactive oxygen species (Lachmandas et al., 2019; Naicker et al., 2020). This combination therapy has also been shown in studies to reduce treatment failure and relapse (Figure 2) (Ma et al., 2018) which also, conflicts with a systematic review done by Yu et al. (2019). It is also linked to the accelerated resolution of cavities observed on chest X-rays, along with a decrease in circulating plasma proinflammatory cytokine levels after an 8-week treatment period in patients with pulmonary TB (Ma et al., 2018; Padmapriydarsini et al., 2022). Singhal et al. (2014) further highlighted metformin’s promise, showing that patients on both metformin and standard anti-TB drugs had better treatment outcomes, with reduced tissue pathology and enhanced immune responses, though these findings stem from preclinical mouse models and *in vitro* macrophage experiments, leaving their human applicability, especially in TB-diabetes (TB-DM) patients, unconfirmed. Furthermore, diabetes primes neutrophils for excessive NET formation through trained immunity, amplifying inflammation and potentially worsening TB pathology (Shrestha et al., 2024). Metformin may counteract this by suppressing proinflammatory cytokines (e.g., IL-1β, TNF-α) and modulating ROS levels, reducing NETosis-driven tissue damage while promoting Mtb clearance.

Despite its adjunctive role in TB management (immunomodulatory and anti-inflammatory properties), some negative effects have been documented. The pharmacokinetics/pharmacodynamics of metformin in combination with standard anti-TB drugs are not yet fully understood. However, some studies have indicated that metformin can influence the plasma levels and clearance rates of key anti-TB medications like rifampicin and isoniazid. Specifically, metformin has been observed to lower the plasma exposure of these drugs and increase their clearance from the body when administered alongside them. This interaction could reduce the efficacy of rifampicin and isoniazid, which are crucial components of the TB treatment regimen (Kumar et al., 2022; Padmapriydarsini et al., 2022). Additionally, rifampicin induces the expression of cytochrome P450 (CYP) enzymes, specifically CYP3A4 and CYP2C9 which accelerates the metabolism of many oral antidiabetic medications, exacerbating hyperglycemia in patients. As a result, patients undergoing TB treatment with rifampicin may require adjustments in their antidiabetic therapy to maintain adequate blood glucose control (Krishna and Jacob, 2000). Also, patients may experience increased stress and anxiety due to the complexity of managing multiple medications and adhering to a strict regimen (Kassaw et al., 2024). This can lead to feelings of overwhelming or frustration, potentially affecting their mental wellbeing. Furthermore, long-term use of metformin has been associated with vitamin B12 deficiency, which can lead to neuropathy and hematologic abnormalities. Since isoniazid also causes peripheral neuropathy, combining metformin with anti-TB drugs could increase the risk of neurological complications. This is particularly concerning for diabetic TB patients who are already prone to diabetic neuropathy. When combined with other antidiabetic medications or during periods of poor nutritional intake (common in TB patients), metformin can increase the risk of hypoglycemia which can be particularly dangerous in TB patients who may already have poor nutritional intake (Xie et al., 2023). In addition, metformin is commonly associated with nausea, vomiting, and diarrhea, which can be exacerbated when combined with TB medications. TB patients already face appetite loss and weight loss, and adding metformin can worsen nutritional deficiencies, further compromising immune function the administration of metformin alongside anti-TB drugs causes nausea and vomiting (Padmapriydarsini et al., 2022).

Incorporating metformin into current TB treatment guidelines is crucial to optimizing its benefits for people with diabetes who have TB. This would include revising the clinical guidelines to incorporate metformin into the usual course of treatment for patients with TBDM and TB only. Endocrinologists and infectious disease experts would need to work together to ensure comprehensive and well-coordinated care to implement such integration. To develop standardized dosage schedules and find any possible interactions with TB drugs in the market, clinical studies and additional research are required (Harries et al., 2016; World Health Organization, 2022a). While metformin shows promise in its adjunctive use in TB treatment the overall quality of evidence is low due to reliance on observational studies, inconsistency in results, methodological limitations, and randomized control trials (RCTs) are insufficient in number and rigor to elevate the certainty beyond low/moderate. Further consistent research is needed to assess its use.

## 4 Future perspectives and research directions

Despite these promising findings, prospective, well-designed clinical trials are essential to validate metformin’s efficacy and safety in patients with TB and DM. Ongoing clinical trials, such as the METHOD trial (U01-AI134585-01A1) and NCT05215990, are evaluating the safety and efficacy of metformin as an adjunct therapy for TB treatment. More trials should be conducted to assess the drug-drug interactions between metformin and the anti-TB drugs, the dosing of metformin and anti-TB medications, the adverse effects of metformin, and the study population (Restrepo, 2016; Yew et al., 2019). Key metrics must be rigorously measured, including sputum conversion rates, time to culture negativity, and post-treatment relapse rates (Singhal et al., 2014). Given that DM can complicate TB treatment and extend recovery times, evaluation of whether metformin enhances the speed and efficacy of TB therapy is critical. Early-phase clinical trials have shown potential benefits, but large-scale, randomized controlled trials are necessary to confirm these findings and establish clinical guidelines (Restrepo, 2016). It is vital to determine whether metformin can mitigate some of these side effects or if it introduces new risks. Monitoring for hepatotoxicity, nephrotoxicity, and gastrointestinal disturbances is essential to ensure that metformin does not exacerbate the common issues associated with anti-TB drugs (Al-Bari et al., 2024). Integrating metformin with existing TB therapies presents a promising strategy to enhance treatment outcomes. This approach is critical given the challenges of poor adherence and developing drug-resistant TB strains associated with prolonged courses of multiple antibiotics in standard TB treatment regimens. Evaluating the efficacy of combination therapies that include metformin is crucial for improving TB management in this patient population. Preliminary studies suggest that metformin may enhance the bactericidal activity of TB drugs, potentially allowing for shorter treatment courses without compromising efficacy (Singhal et al., 2014). Moreover, metformin’s anti-inflammatory properties could mitigate common side effects of TB medications, such as hepatotoxicity and tissue damage, improving treatment tolerability (Singhal et al., 2014).

Exploring the mechanisms of metformin in TB control is a promising area of future research. One potential direction is to investigate how metformin modulates the host immune response to Mtb infection. Understanding these pathways in greater detail could reveal new therapeutic targets for TB treatment (Yew et al., 2020). Additionally, research should focus on integrating metformin with existing anti-TB therapies. Investigating the potential synergistic effects of metformin and standard TB medications could significantly advance TB therapies (Sutter et al., 2022). Studies should explore whether metformin can reduce the duration of TB therapy without compromising efficacy, improving patient compliance, or reducing the risk of drug resistance (Naicker et al., 2020). Metformin’s dual role controlling blood glucose while enhancing immunity offers a compelling strategy, as improved glycemic control has been linked to better pulmonary outcomes and immune responses in TB-DM patients (Sutter et al., 2022; Sutter et al., 2024).

Optimizing metformin’s dosing and administration in TB treatment is another critical research avenue. Pharmacokinetic studies in TB-DM patients are needed to develop effective protocols, ensuring therapeutic benefits are maximized (Kumar et al., 2022; Restrepo, 2016). By delving into metformin’s immune-modulating mechanisms, integrating it with existing therapies, and confirming its clinical utility, researchers can unlock innovative strategies to improve TB management. This approach holds particular promise for enhancing outcomes in the challenging TB-DM population, paving the way for more effective, tolerable, and efficient treatment regimens (Singhal et al., 2014; Sutter et al., 2022).

While this review synthesizes critical insights into metformin’s potential role in TB-DM management, its non-systematic approach introduces limitations, including potential selection bias from unstructured methodology, lack of study quality appraisal, and reliance on narrative synthesis over quantitative analysis. Without systematic evidence grading, clinical recommendations remain speculative. Future work should adopt systematic review guidelines to enhance rigor and validity.

## 5 Conclusion

The integration of metformin into TB treatment regimens for patients with DM may hold significant promise for treatment outcomes. Research on metformin as an adjunct therapy for TB treatment often encounters significant variability in dosing and combination therapies, impacting the results’ precision and applicability (Singhal et al., 2014). Also, many research focus on only diabetic individuals, limiting the generalizability of findings to non-diabetic TB patients and those with other comorbidities like HIV or cardiovascular diseases (Cáceres et al., 2022; Padmapriydarsini et al., 2022). Therefore, future research should focus on determining the optimal dosing strategies for metformin in TB treatment, understanding its pharmacokinetics in TB patients, and exploring its potential interactions with existing anti-TB medications (Kumar et al., 2022; Singhal et al., 2014; Sutter et al., 2022). Bridging these gaps will help translate research findings into broader clinical applications, benefiting a wider range of patients.

## Author contributions

PT: Writing – original draft, Writing – review and editing. ED: Writing – review and editing. SO-W: Writing – review and editing. DY-M: Funding acquisition, Resources, Supervision, Validation, Writing – review and editing. PA: Supervision, Validation, Writing – review and editing.
